# Bibliometric analysis of rheumatic immune related adverse events associated with immune checkpoint inhibitors

**DOI:** 10.3389/fimmu.2023.1242336

**Published:** 2023-10-06

**Authors:** Li Zeng, Gang Ma, Kai Chen, Qiao Zhou

**Affiliations:** ^1^ Department of Neurology, Sichuan Provincial People’s Hospital, University of Electronic Science and Technology of China, Chengdu, China; ^2^ Chengdu University of Traditional Chinese Medicine, Chengdu, China; ^3^ Department of Rheumatology and Immunology, Sichuan Provincial People’s Hospital, University of Electronic Science and Technology of China, Chengdu, China; ^4^ Clinical Immunology Translational Medicine Key Laboratory of Sichuan Province, Sichuan Provincial People’s Hospital, University of Electronic Science and Technology of China, Chengdu, China

**Keywords:** immune checkpoint inhibitor, rheumatic immune-related adverse event, bibliometric, research trends, VOSviewer, CiteSpace

## Abstract

**Background:**

Immune checkpoint inhibitors (ICIs) has emerged as a popular cancer treatment approach. However, non-specific activation of T cells by ICIs can lead to immune-related adverse events (irAEs), including specific rheumatic manifestations. The study aimed to explore the current trend of ICIs associated rheumatic irAEs and summarize the knowledge structure through bibliometric methods.

**Methods:**

The Web of Science Core Collection database (WoSCC) was selected for retrieving literature on ICIs associated rheumatic irAEs. To evaluate contributions from different countries/regions, institutions, journals, and authors, bibliometric analysis software, including VOSviewer and CiteSpace, as well as bibliometric online platforms, were utilized to construct and visualize bibliometric networks. Through the systematic review of this knowledge domain, future research directions were determined.

**Results:**

In This study, a total of 803 publications on ICIs-associated rheumatic irAEs were included for analysis. The distribution of these publications revealed two distinct growth phases: a stable phase between 2007 to 2015 followed by rapid growth from 2016 to 2020. The United States emerged as the top contributor in terms of publications, citations, and h-index, with the majority of leading institutions and funding agencies located there. Apart from government funding, pharmaceutical companies such as Bristol Myers Squibb and Merck Company also play a significant role in drug development and research. Analysis of keywords and citation bursts indicated that the initial burst was related to “monoclonal antibody,” “anti-CLTA4 antibody,” and “melanoma”. This was followed by a rise in interest related to “sarcoidosis,” “safety,” “inflammatory arthritis,” and “preexisting autoimmune.”

**Conclusion:**

This study summarized the global research trends concerning ICIs associated rheumatic irAEs. The findings can provide valuable insights into the current understanding of rheumatic irAEs, highlight the research trend and developments in the field. Future efforts should focus on developing classification criteria and guidelines, conducting prospective studies, investigating the mechanisms involved, and identifying biomarkers for prediction and monitoring of these events.

## 1 Introduction

In recent years, immunotherapy has become increasingly common in cancer treatment, as they have shown remarkable success in inducing durable responses in some patients (1). Monoclonal antibodies targeting immunological checkpoints, or immune checkpoint inhibitors (ICIs), represent a rapidly growing class of these agents. By targeting the T-cell cytotoxic T-lymphocyte-associated protein 4 (CTLA-4) or the programmed cell death-ligand 1 (PD-1/PD-L1) coinhibitory receptors, ICIs, either as single agents or in combination, enhance antitumor T-cell activity, leading to unprecedented long-lasting tumor responses in patients with unresectable or advanced metastatic disease (2–6).

However, while these drugs non-specifically activate T cells, which can not only increase immune response against cancer cells, but also lead to immune-related adverse events (irAEs) that exhibit inflammatory or autoimmune-like side effects. These adverse events (AEs) are unique in comparison to conventional cancer therapies and can range from mild to severe, affecting any organ system in the body (7, 8). The incidence of irAEs depends on various factors, such as the type of ICIs used, the cancer type, and the individual patient’s characteristics. Common irAEs associated with ICIs include pneumonitis, colitis, hepatitis, endocrinopathies, and skin toxicities (9). Interestingly, specific rheumatic manifestations among irAEs have also been described, such as inflammatory arthritis, vasculitis, inflammatory myopathy, sicca syndrome, and scleroderma, etc (9). However, the prevalence and course of these rheumatic irAEs are not well-established, and detecting them during or after cancer treatment can be challenging, as they resemble conventional rheumatologic diseases (10). Clinical trials often overlook musculoskeletal/rheumatic events as a distinct organ system or report only high-grade and/or frequent adverse events occurring in at least 10% of the patients (11). Additionally, different researches might use varying terms to describe these irAEs due to their heterogeneous definitions, and real-life data suggest that they are underreported (12, 13). Also, evidence is lacking for the optimal diagnostic approach and management of these patients while allowing effective antitumor therapy to continue (9). Consequently, it is crucial to gain a comprehensive understanding of the current status, research trends and emerging topics.

Bibliometric analysis is a useful tool for achieving this goal, as it allows for the systematic and quantitative analysis of research publications in a particular field. No such studies have been published in the field of ICIs associated rheumatic irAEs. By using bibliometric analysis techniques, we aim to provide an overview of the studies and scholarly contributions, identify the most influential authors, institutions, and journals in this field and to map the most significant research topics and trends, which can contribute to the improvement of patient care and the development of safer immunotherapies. Furthermore, the results of this study can also inform policymakers and funding agencies about the most promising areas of research in the field of immunotherapy-related adverse events.

## 2 Methods

### 2.1 Data sources and retrieval strategy

**Figure 1:**
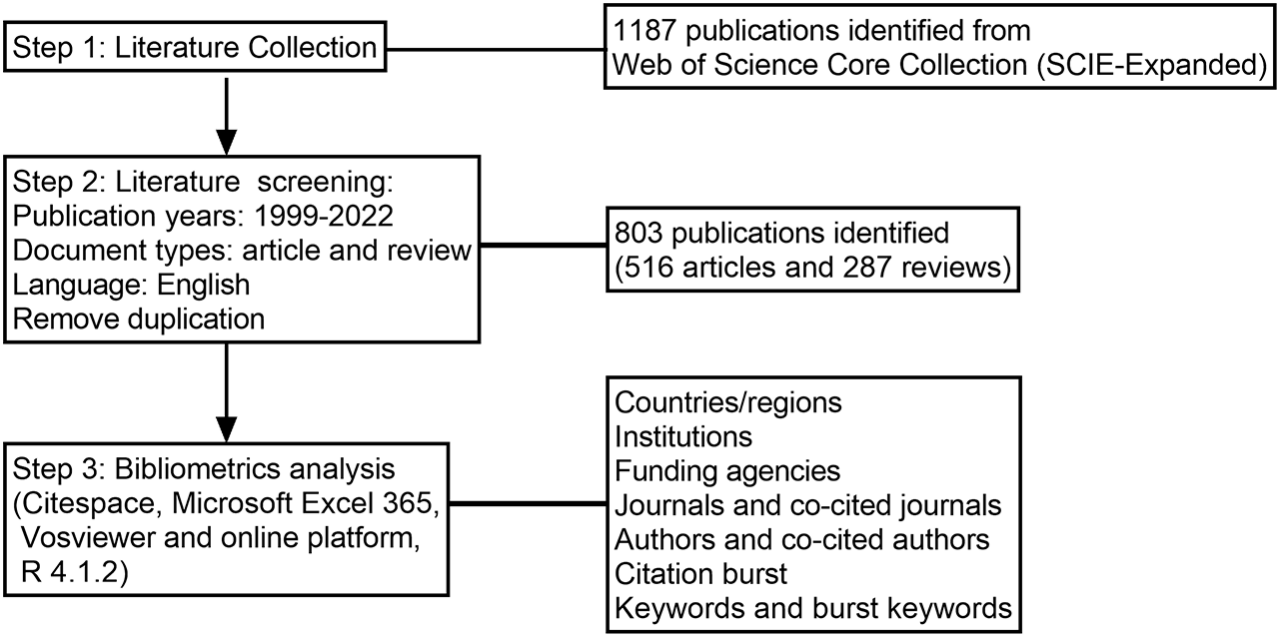
The CONSORT diagram of the research process.

The bibliographic data were all obtained from the Science Citation Index Expanded (SCI-expanded, 1999-present) of the Web of Science Core Collection (WoSCC). Rheumatic irAEs were identified through literature review (9, 11, 14). To perform a comprehensive literature search of ICIs associated rheumatic irAEs, specific retrieval rules were designed as follows: #1: TS=(ipilimumab) or TS=(avelumab) or TS=(nivolumab) or TS=(atezolizumab) or TS=(pembrolizumab) or TS=(durvalumab) or TS=(cemiplimab) or TS=(relatimab) or TS=(“immune checkpoint inhibitor*”) or TS=(“immune checkpoint block*”) or TS=(“immune checkpoint therap*”). #2: TS=(vasculitis) or TS=(arteritis) or TS=(polyangiitis) or TS=(lupus) or TS=(“systemic sclerosis”) or TS=(scleroderma) or TS=(“skin thickening”) or TS=(raynaud*) or TS=(erythema nodosum) or TS=(sarcoidosis) or TS=(sarcoid-like) or TS=(“sicca symptoms”) or TS=(“dry mouth”) or TS=(parotitis) or TS=(“Sjögren’s syndrome”) or TS=(enthesitis) or TS=(dactylitis) or TS=(tenosynovitis) or TS=(synovitis) or TS=(“musculoskeletal pain”) or TS=(myalgia) or TS=(myositis) or TS=(dermatomyositis) or TS=(arthritis) or TS=(arthralgia) or TS=(*arthropathy) or TS=(“polymalgia rheumatica”) or TS=(rheumatic) or TS=(“connective tissue disease”). #3: #1 and #2. The time span of the search was from 1999 to 2022 (retrieved on November 24, 2022), and a total of 1187 records were collected from WoSCC. In the end, 803 ultimate documents were remained after excluding non-English literature and restricting document types to articles and reviews. The search details were shown in **Figure 1**.

### 2.2 Data analysis and result visualization

We conducted data analysis and result visualization using multiple tools, including Citespace (Version 5.6.R2), VOSviewer (Version 1.6.18), and an online platform (https://bibliometric.com/), as well as two R packages, Bibliometrix and Biblioshiny (Version 4.1.2).

CiteSpace is a Java-based visualization tool that offers a range of visualization options, such as network maps, centrality and burst maps, which reveal clusters and dynamics related to a specific scientific topic (15). The centrality of nodes is calculated to reveal their importance within the network (16). The burst maps were applied to detect keywords and references, which recognizes sudden significant increases in a scientific activity over a certain period (17). VOSviewer is another Java-based bibliometric analysis software that allows for the exploration and visualization of research characteristics from various perspectives, providing three types of network maps: the network visualization map, the overlay visualization map, and the density visualization map (15). In the network visualization map, each node corresponds to parameters, such as countries/regions, institutions, journals, authors, or keywords. The node size represents its strength (e.g., the number of publications, citations, and occurrences) while the thickness of the link represents the strength of the network associations. The color-coded nodes and links represent clusters and connections, respectively, and clusters with the same color are automatically assigned to closer terms (18). The python pyechats was used to build the world map, while an online bibliometric analysis platform was applied to visualize the distribution and international cooperation among countries/regions. The different tools used in this study allowed for a comprehensive and nuanced analysis of the data and provided valuable insights into the current status, knowledge base, research trends, and emerging topics in the field of rheumatic irAEs associated with ICIs.

## 3 Results

### 3.1 Distribution of publication outputs and citations by year

**Figure 2:**
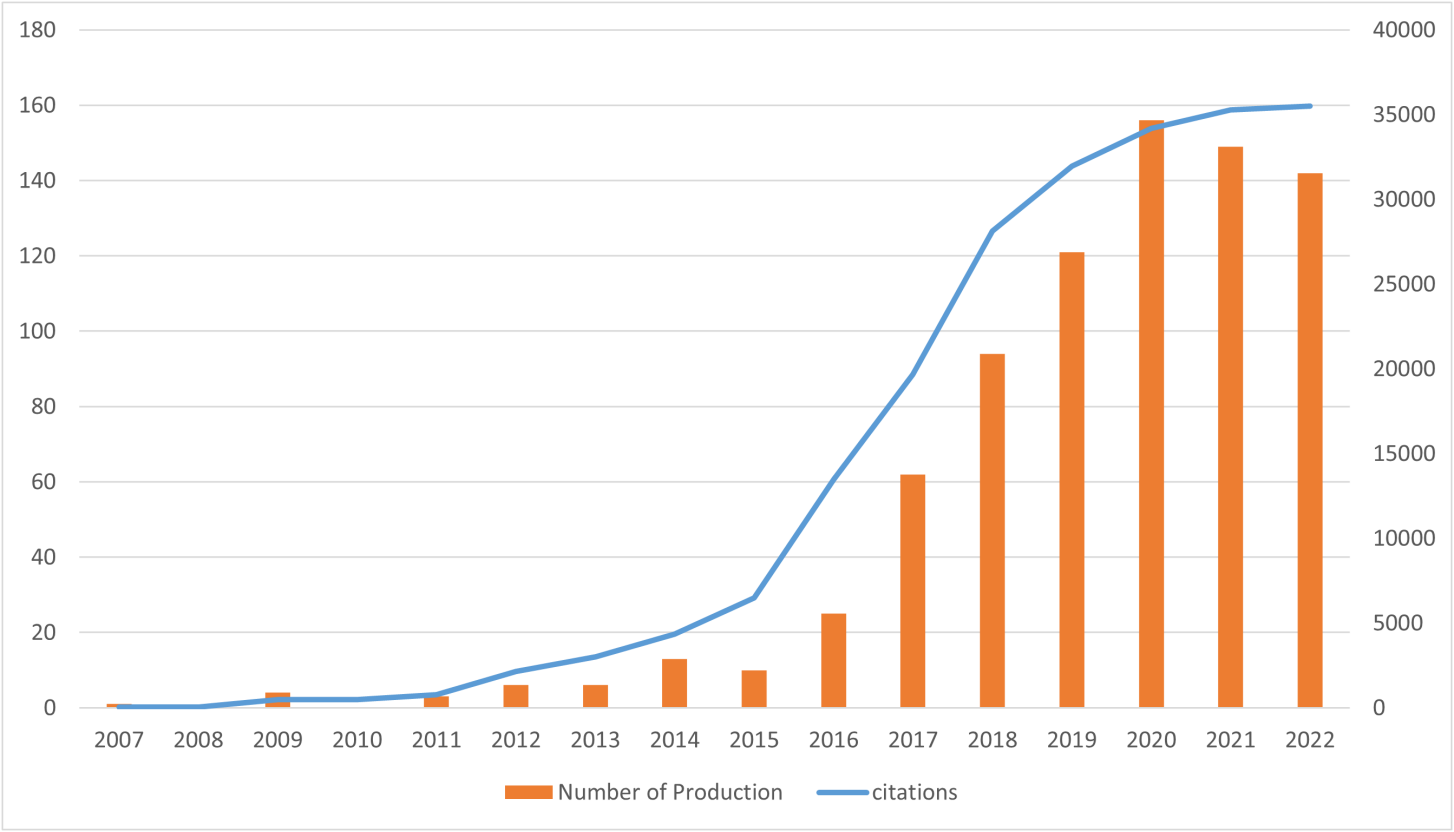
Publication trend and citation analysis of ICIs associated rheumatic irAEs worldwide.

Performing a quantitative analysis of publications in a specific field can provide valuable insights into academic hotspots and trends. In this study, we identified 803 publications, including 516 articles and 287 reviews, through the screening process illustrated in **Figure 1**. The first literature, which was a review article, on ICIs associated rheumatic irAEs was published in the United States of America (USA) in 2007, following the approval of the CTLA4 blockade (**Figure 2**) (19). The number of publications per year has steadily increased over the past 16 years, with occasional dips, and peaked in 2020, with two distinct growth phases: a stationary phase from 2007 to 2015, followed by a rapid growth phase from 2016 to 2020 (**Figure 2**). Of the 803 documents, 82.44% were published within the last 5 years, with a total citation count of 35,506 times and an average of 44.22 citations per paper. As ICIs have become more popular in recent years, rheumatic irAEs have received greater attention.

### 3.2 Country/region specific analyses of published articles

**Figure 3:**
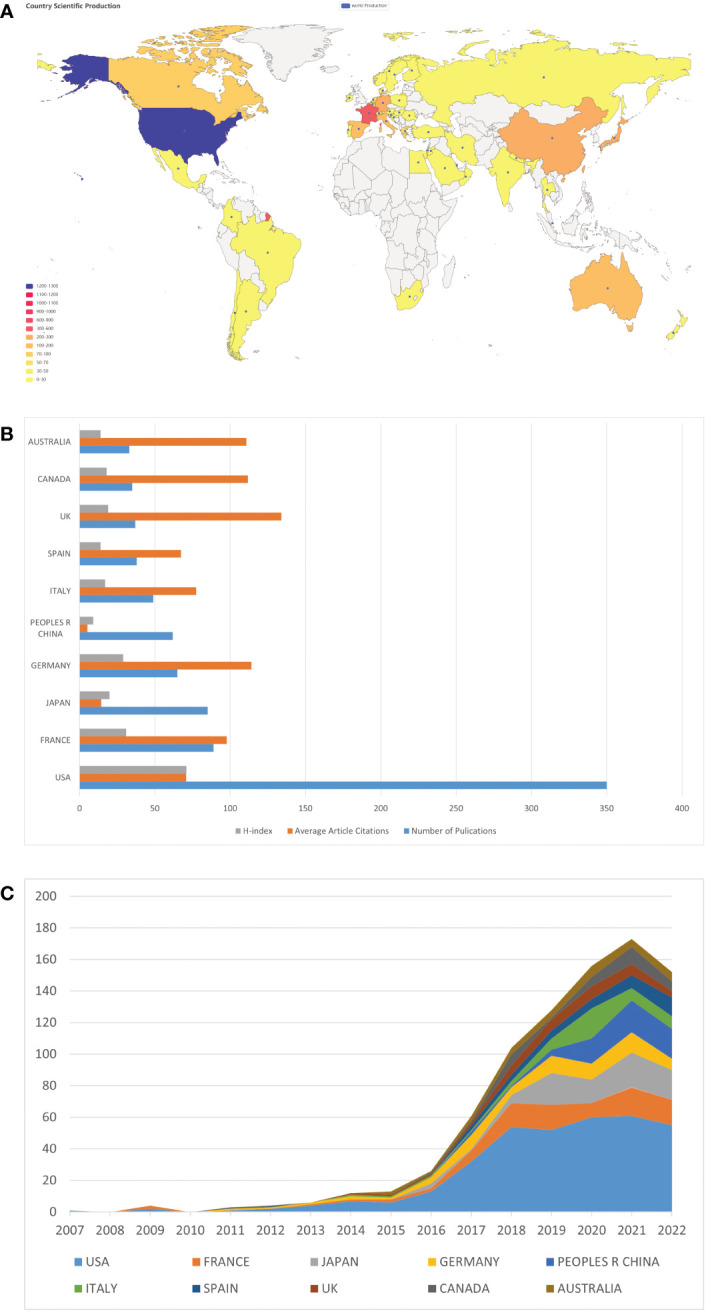
Country/region specific analyses of publications on ICIs associated rheumatic irAEs. **(A)** Geographic distribution of global publications. The color gradient in the visualization represents the number of publications, where the darker color indicates a higher number of publications while the lighter color represents a lower number of publications. **(B)** Number of publications, average citations per article, and H-index of the top 10 countries/regions. **(C)** Comparison of the number of publications by the top 10 countries/regions between 2007 and 2022.

We then analyzed the geographical distribution of the 803 retrieved articles and identified 52 countries/regions as the origins. The spatial distribution heatmap showed higher publication density in darker color and lower publication density in lighter color (**Figure 3A**). The USA ranked first among the top ten countries with 350 publications, followed by France (89), Japan (85), Germany (65), and China (62) (**Table 1** and **Figure 3B**). A detailed analysis of citations and H-index across all countries/regions revealed that the USA had the most citations (24,786) and the highest H-index (71). Notably, Japan and China had relatively high numbers of publications, but their citation counts (1,218 and 328, respectively) and H-indices (20 and 9, respectively) were lower, indicating a need for improvement in the quality of their publications. By contrast, Germany, the United Kingdom (UK), Canada, and Australia have relatively lower publication output but higher citation rates (all exceeds 100 citations per article) compared to other countries, suggesting that the research they do publish is of high quality and has a significant impact on the field (**Table 1** and **Figure 3B**). The USA still ranked first in the number of publications per year compared to other countries (**Figure 3C**). An analysis of international collaboration between countries showed that the USA had the most collaborations (mainly with France, Australia, and Italy), followed by France (**Supplementary Figure 1A**). We also performed a network visualization analysis using VOSviewer, which included countries/regions with at least five publications. The resulting network map consisted of 25 nodes, 7 clusters, and 273 links, with the USA located at the center of the node, indicating its significant contributions to research in this field (**Supplementary Figure 1B**).

**Table 1:** The top 10 most productive countries and regions for ICI-related rheumatological side effects.

Rank	Country/Region	Number of Publications	Number of Citations	Citations per article	H-Index
1	USA	350	24786	70.82	71
2	FRANCE	89	8696	97.71	31
3	JAPAN	85	1218	14.33	20
4	GERMANY	65	7412	114.03	29
5	PEOPLES R CHINA	62	328	5.29	9
6	ITALY	49	3794	77.43	17
7	SPAIN	38	2560	67.37	14
8	UK	37	4955	133.92	19
9	CANADA	35	3912	111.77	18
10	AUSTRALIA	33	3658	110.85	14

### 3.3 Analysis of leading institutions

**Figure 4:**
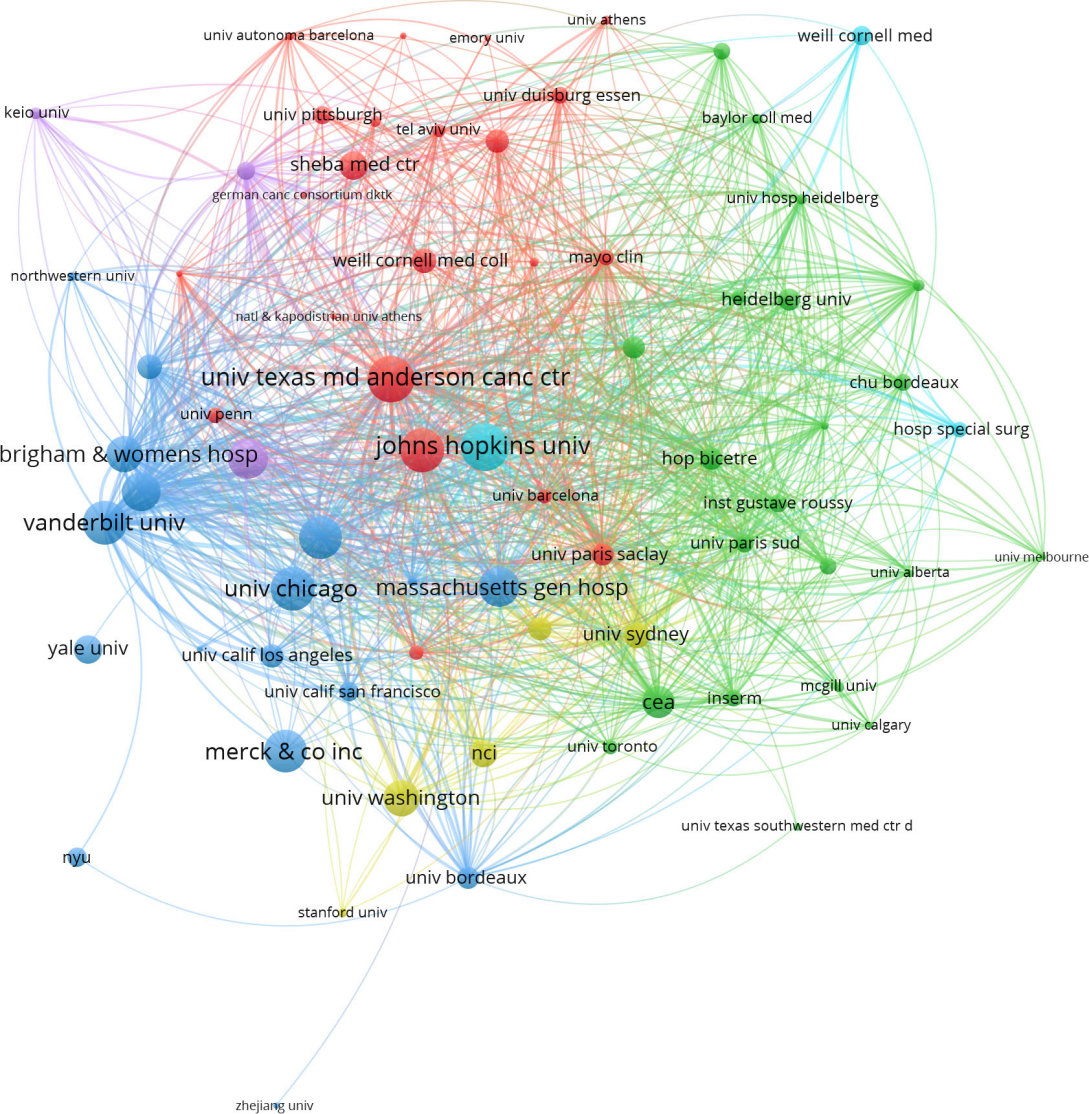
Co-citation analysis of institution using VOSviewer. In the network visualization, nodes represent institutions, and their size reflects the number of publications. Lines between nodes indicate co-citation relationship.

After removing duplicates and irrelevant organizations, we found 1354 institutions with publications related to ICIs associated rheumatic irAEs. The top 10 most productive institutions accounted for 63.14% (507 publications) of the total output, with Udice France Research University (63 publications), University of Texas System (57 publications), and Harvard University (56 publications) being the top three (**Table 2**). Six of the top 10 institutions came from the USA, and four came from France. Additionally, we visualized the institution citation analysis using VOSviewer, which included institutions with at least five publications (**Figure 4**). The resulting network map contained 73 nodes, 6 clusters, and 2027 links, with the University of Texas MD Anderson Cancer Center being the central node. This indicated that these institutions are key players in advancing research on ICIs associated rheumatic irAEs.

**Table 2:** A list of the top ten institutions with the most publications on ICI-related side effects in rheumatology.

Rank	Institution	Country	Number of Publications	Number of Citations	Citations per article	Percentage (N=802)	H-Index
1	UDICE FRENCH RESEARCH UNIVERSITIES	France	63	6437	102.17	7.855	29
2	UNIVERSITY OF TEXAS SYSTEM	USA	57	5756	100.98	7.107	23
3	HARVARD UNIVERSITY	USA	56	101077	1804.95	6.983	29
4	ASSISTANCE PUBLIQUE HOPITAUX PARIS APHP	France	51	4703	92.22	6.359	26
5	INSTITUT NATIONAL DE LA SANTE ET DE LA RECHERCHE MEDICALE INSERM	France	49	4457	90.96	6.11	24
6	UTMD ANDERSON CANCER CENTER	USA	49	5590	114.08	6.11	22
7	JOHNS HOPKINS UNIVERSITY	USA	39	7118	182.51	4.863	23
8	HARVARD MEDICAL SCHOOL	USA	30	2819	93.97	3.741	26
9	SORBONNE UNIVERSITE	France	28	1789	63.89	3.491	19
10	DANA FARBER CANCER INSTITUTE	USA	27	6322	234.15	3.367	19

### 3.4 Analysis of top funding agencies

Funding support is crucial for the advancement of any research field, as it attracts more researchers and institutions to dedicate their work to the area. Therefore, we identified the top 10 funding agencies that have provided crucial support to the field of ICIs associated rheumatic irAEs research (**Table 3**). Notably, the top five agencies are all from the USA, comprising of three research institutions, namely the USA Department of Health Human Services (HHS), National Institutes of Health (NIH), and NIH National Cancer Institute (NCI), as well as two companies, Bristol Myers Squibb and Merck Company. This finding highlights the significant financial support that the USA provides to this research field, thereby maintaining its leading position. The remaining funding agencies are from China, Japan, and Switzerland (**Table 3**).

**Table 3:** A list of the top 10 funding agencies contributed to rheumatological side effects related to ICIs.

Rank	Funding agencies	Country	Count	Percentage (N=802)	H-Index
1	United States Department of Health Human Services (HHS)	USA	88	12.594	36
2	National Institutes of Health (NIH)	USA	87	12.469	36
3	Bristol Myers Squibb	USA	38	6.608	24
4	NIH National Cancer Institute (NCI)	USA	36	4.613	19
5	Merck Company	USA	24	2.993	15
6	National Natural Science Foundation of China (NSFC)	China	20	2.494	6
7	NIH National Institute of Arthritis Musculoskeletal Skin Diseases (NIAMS)	USA	17	2.369	11
8	Japan Society for The Promotion of Science (JSPS)	Japan	12	1.496	8
9	Ministry Of Education Culture Sports Science and Technology Japan (MEXT)	Japan	12	1.496	8
10	Roche Holding	Switzerland	12	1.372	8

### 3.5 Analysis of top journals and co-cited journals

**Figure 5:**
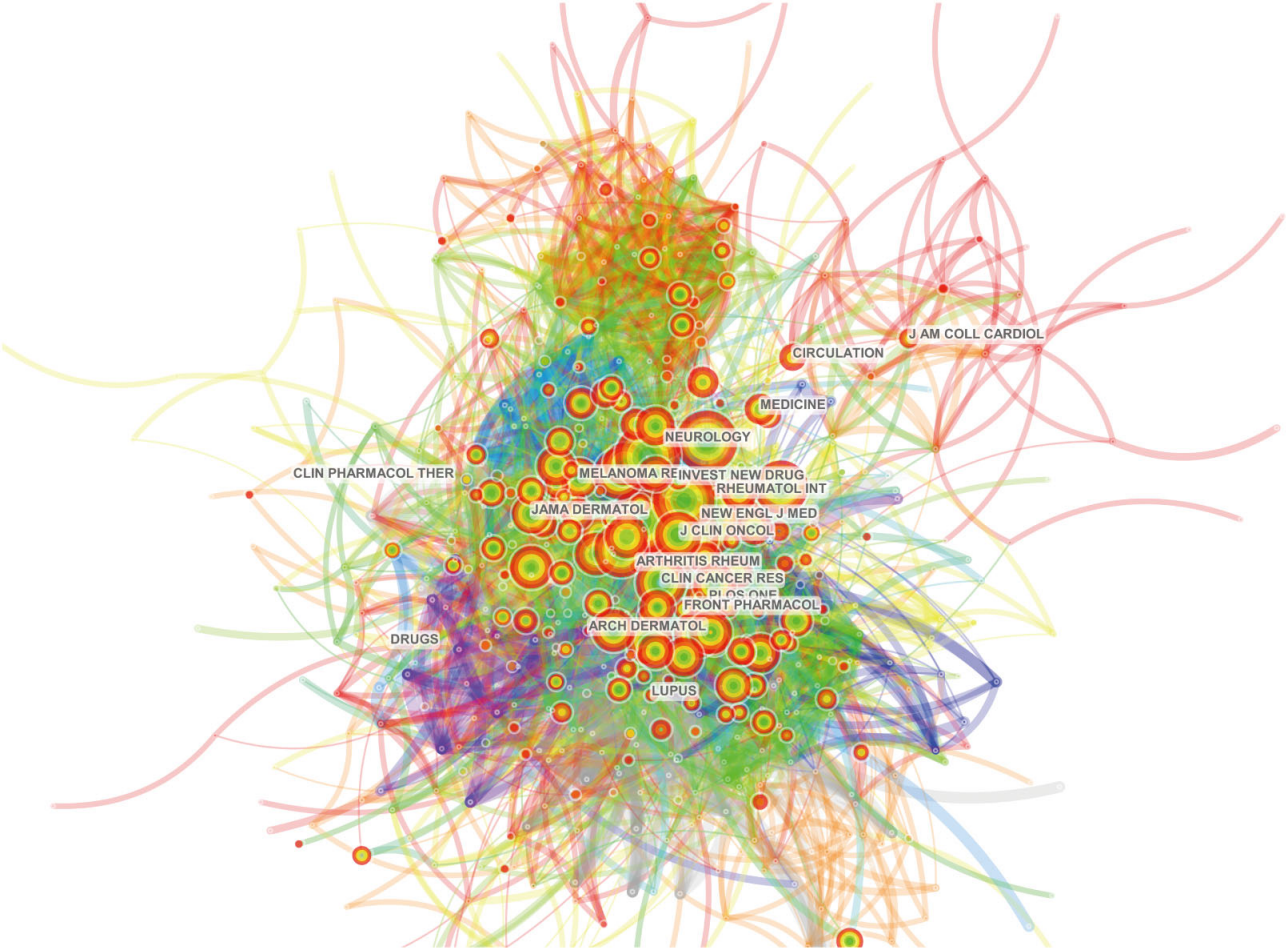
Journal co-citation analysis in the field of ICIs associated rheumatic irAEs using CiteSpace.

Our bibliometric analysis identified 316 journals that have published articles on ICIs associated rheumatic irAEs, with the top 10 journals publishing 190 publications (23.66%) (**Table 4**). *The Journal for ImmunoTherapy of Cancer* had the highest number of publications (38), with 2259 citations and the highest h-index (22) (**Table 4**). Notably, *the European Journal of Cancer* had only 14 publications but the highest number of citations (2355), resulting in the highest citations per article (168.21). Both journals are classified as Q1 in the JCR partition. *Frontiers in Immunology* and *Frontiers in Oncology* had a relatively high number of publications (25 and 20, respectively), but their total citations were relatively low, and they were classified as Q1 and Q2 categories in the JCR partition (**Table 4**). We used Citespace software to analyze connections between journals cited in other journals and found a co-citation network map with 578 nodes and 4913 links (**Figure 5**). Centrality measurements revealed that no particular journal had high centrality or was highly cited in other journals (**Supplementary Figure 2**). Additionally, we conducted a dual map overlay of journals related to ICIs associated rheumatic irAEsand found that published studies focused on two field: molecular biology and immunology, and medicine, medical, and clinical (**Supplementary Figure 3**). The most cited publications originated from journals in the fields of molecular biology and genetics, and health, nursing, and medicine.

**Table 4:** A list of the top 10 journals contributed to rheumatological side effects related to ICIs.

Rank	Journal	Country	JCR partition	Impact Factor	Number of publications	Number of citations	Citations of per article	Percentage (N=802)	H-Index
1	JOURNAL FOR IMMUNOTHERAPY OF CANCER	USA	Q1	12.469	38	2259	59.45	4.613	22
2	FRONTIERS IN IMMUNOLOGY	Switzerland	Q1	8.786	25	301	12.04	3.117	9
3	FRONTIERS IN ONCOLOGY	Switzerland	Q2	5.738	20	99	4.95	2.494	6
4	JOURNAL OF IMMUNOTHERAPY	USA	Q2	4.912	20	395	19.75	2.494	9
5	MELANOMA RESEARCH	USA	Q2	3.199	17	261	15.35	1.995	8
6	ONCOLOGIST	USA	Q2	5.837	16	603	37.69	1.995	9
7	EUROPEAN JOURNAL OF CANCER	England	Q1	10.002	14	2355	168.21	1.746	11
8	IMMUNOTHERAPY	England	Q3	4.040	14	144	10.29	1.746	6
9	AUTOIMMUNITY REVIEWS	USA	Q1	17.39	13	381	29.31	1.621	10
10	CANCER IMMUNOLOGY IMMUNOTHERAPY	USA	Q1	6.63	13	734	56.46	1.621	10

### 3.6 The top productive authors

The bibliometric analysis of the 803 publications identified 5106 authors as contributors. **Table 5** presents the top 10 most productive authors in the field, led by Suarez-Almazor ME with 21 papers and closely followed by Johnson DB with 18 publications. Notably, Robert C achieved the highest total citations with 4287, despite only having 11 publications in the field, averaging 389.73 citations per article. This highlights their valuable and impactful research contributions to the field.

**Table 5:** A list of the top 10 authors contributed to rheumatological side effects related to ICIs.

Rank	Author	Country	Number of publications	Number of citations	Citations of per article	Percentage (N=802)	H-Index
1	Suarez-almazor ME	USA	21	4141	197.19	2.618	13
2	Johnson DB	USA	18	991	55.06	2.244	14
3	Cappelli LC	USA	16	2459	153.69	1.995	10
4	Lambotte O	France	16	3074	192.13	1.995	11
5	Abdel-wahab N	USA	14	911	65.07	1.746	11
6	Bingham CO	USA	13	2001	153.92	1.621	10
7	Moslehi JJ	USA	13	1036	79.69	1.621	11
8	Bass AR	USA	12	852	71	1.496	7
9	Hassel JC	Germany	11	846	76.91	1.372	8
10	Robert C	France	11	4287	389.73	1.372	8

### 3.7 References and citation burst

**Figure 6:**
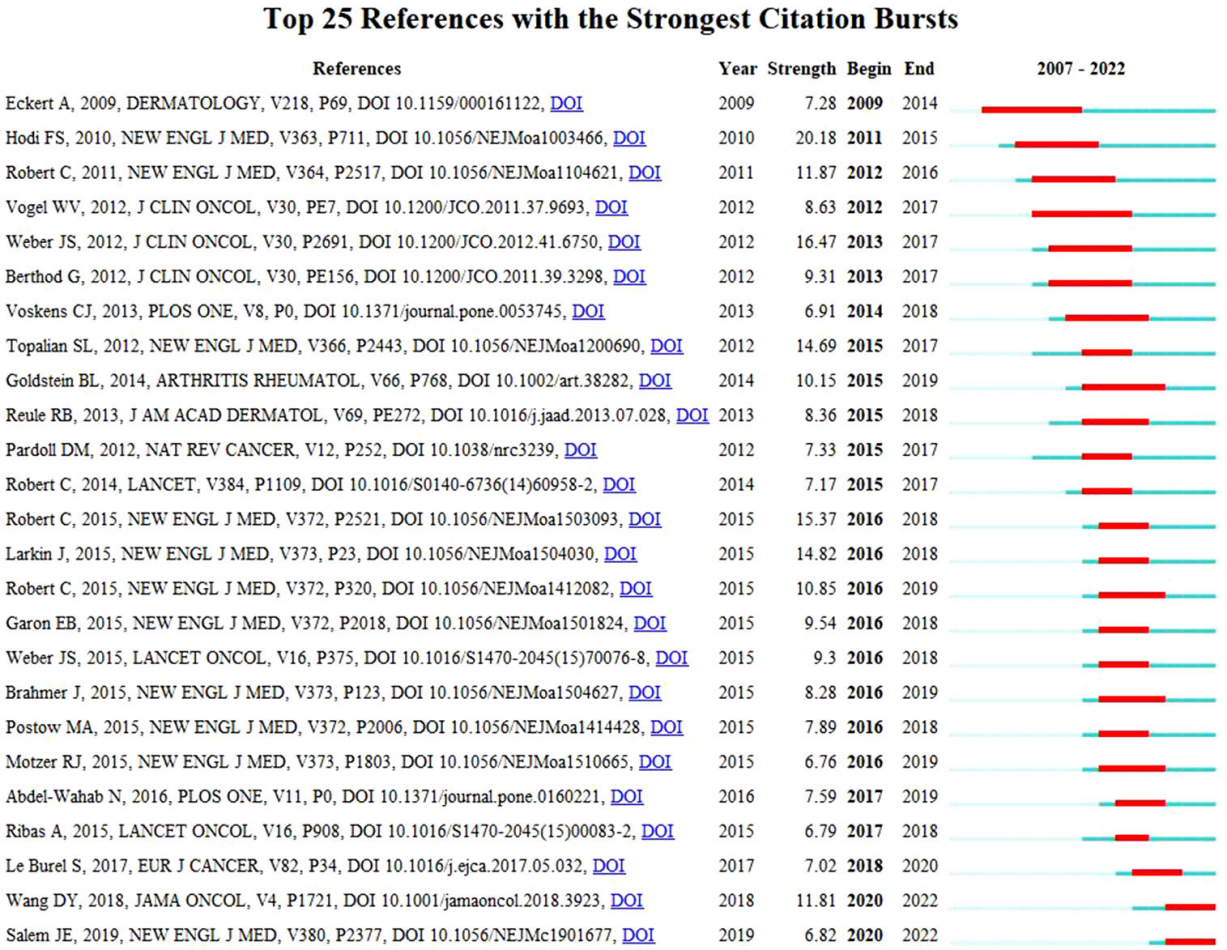
List of the 25 references with the strongest citation bursts.

To identify research frontiers and emerging trends in this field, we analyzed the top 10 most cited publications and citation burst (**Table 6**, **Figure 6**). **Table 6** summarized the top 10 most cited publications in the field of ICIs associated rheumatic irAEs, covering the period from 2012 to 2018. The publication by Brahmer JR, published in 2018, received the highest number of citations, with 1893 citations, followed by Nanda R, with 1334 citations. Michot, JM published the third co-cited paper with 1304 citations. Subsequently, we applied burst detection in Citespace to identify research hotspots over time for references. **Figure 6** displays the top 25 references with the most robust citation bursts, with the red line indicating the duration of the reference burst occurrence. The first burst of the co-cited reference was a case report that initially reported the induction of sarcoidosis during the treatment of metastatic melanoma with anti-CTLA4 monoclonal antibody, beginning in 2009 and ending in 2014 (20). Currently, two references are still in the burst, focusing mainly on ICIs associated fatal toxic effects and myocarditis. Thus, ICIs associated side effects will be further explored in the future (21, 22).

**Table 6:** A list of the top 10 co-cited references.

Rank	Title	Journal	Author	Years	Citations
1	Management of Immune-Related Adverse Events in Patients Treated With Immune Checkpoint Inhibitor Therapy: American Society of Clinical Oncology Clinical Practice Guideline	Journal of Clinical Oncology	Brahmer, JR	2018	1893
2	Pembrolizumab in Patients With Advanced Triple-Negative Breast Cancer: Phase Ib KEYNOTE-012 Study	Journal of Clinical Oncology	Nanda, R	2016	1334
3	Immune-related adverse events with immune checkpoint blockade: a comprehensive review	European Journal of Cancer	Michot, JM	2016	1304
4	Fulminant Myocarditis with Combination Immune Checkpoint Blockade	New England Journal of Medicine	Johnson, DB	2016	1199
5	Pembrolizumab versus investigator-choice chemotherapy for ipilimumab-refractory melanoma (KEYNOTE-002): a randomised, controlled, phase 2 trial	Lancet Oncology	Ribas, A	2015	1140
6	Managing toxicities associated with immune checkpoint inhibitors: consensus recommendations from the Society for Immunotherapy of Cancer (SITC) Toxicity Management Working Group	Journal for Immunotherapy of Cancer	Puzanov, I	2017	1066
7	Adjuvant Pembrolizumab versus Placebo in Resected Stage III Melanoma	New England Journal of Medicine	Eggermont, AMM	2018	1036
8	Management of Immune-Related Adverse Events and Kinetics of Response With Ipilimumab	Journal of Clinical Oncology	Weber, JS	2012	1020
9	Avelumab in patients with chemotherapy-refractory metastatic Merkel cell carcinoma: a multicentre, single-group, open-label, phase 2 trial	Lancet Oncology	Kaufman, HL	2016	816
10	First-line pembrolizumab in cisplatin-ineligible patients with locally advanced and unresectable or metastatic urothelial cancer (KEYNOTE-052): a multicentre, single-arm, phase 2 study	Lancet Oncology	Balar, AV	2017	774

### 3.8 Keywords and citation burst

In addition to references, keywords can provide valuable insights into the research hotspots and directions of a specific research field. With the help of VOSviewer, we conducted a visual analysis of keywords to generate the visualization map based on keywords with a co-occurrence value above 20. **Supplementary Figure 4A** highlights closely related keywords grouped into three clusters, with 149 nodes, 9328 links, and a total link strength of 44142. “ICIs” was at the center of node in the red cluster, with 148 links and a link strength of 1958. “Nivolumab,” the center of node for the green cluster, has 147 connections and a total link strength of 1503. “Effect,” the center of node for the blue cluster, has 148 connections and a total link strength of 2328. **Supplementary Figure 4B** presents the density visualization map of keywords, with “effect,” “ICIs” and “nivolumab” as the top three keywords, appearing 228, 179 and 136 times, respectively. **Supplementary Figure 4C** demonstrates a time-lapse visualization map, with purple representing earlier dates and yellow representing more recent dates. “ipilimumab” was the top keyword in 2017, appearing 127 times. “Effect” was the top keyword in 2019, appearing 228 times, and “ICIs” in 2020, appearing 179 times. The pattern is consistent with drug research norms, in which researchers examine the effects of a drug before evaluating its long-term side effects. Finally, we applied burst detection in Citespace to identify research hotspots over time for keywords. **Supplementary Figure 4D** shows that the first burst of keywords included “monoclonal antibody,” “anti-CLTA4 antibody,” and “melanoma”. Subsequently, induced sarcoidosis (2012-2018, strength of 3.51), safety (2015-2017, strength of 3.48), inflammatory arthritis (2018-2019, strength of 3.43), and preexisting autoimmune (2019-2020, strength of 3.51) garnered increasing attention from researchers.

## 4 Discussion

In this study, we conducted a bibliometric analysis of publications related to ICI-induced rheumatic irAEs for the first time to gain insights into the global research trends in this field. In contrast to scoping reviews and systematic reviews, bibliometric analysis provides a powerful tool for summarizing current knowledge and identifying emerging trends. After excluding 384 publications that didn’t meet our inclusion criteria, we identified 803 publications in 316 journals from 1354 institutions in 52 countries/regions. The first article was published in 2007, describing the effectiveness of CTLA-4 inhibitors in Phase I/II clinical trials. It was also observed that some patients developed arthritis, and showed improvement after treatment with Cox-2 inhibitors. A significant increase in publications related to this topic over the years was revealed, particularly since 2015, indicating the growing importance of this field of research. One possible explanation for this trend is the increasing clinical use of ICIs since the first US Food and Drug Administration (FDA) approval of ipilimumab for late-stage (metastatic) melanoma in 2011 (23). The approval of several ICIs in 2015 for various types of cancer (24) led to a surge in their use and a subsequent increase in the reports of rheumatic irAEs. As more patients were treated and more AEs were reported, the interest and awareness of this topic increased among the research community.

The distribution of contributing countries/regions and institutions showed some characteristics. The USA was the most productive country in terms of publications, followed by France and Japan. The top 10 institutions accounted for 55.9% of the papers in this field, highlighting the significant contributions made by researchers from these countries. The USA’ dominant position can be attributed to its well-established research infrastructure, long history of medical research, top-ranked institutions, and funding agencies such as the National Institutes of Health (NIH), which is one of the most important research funding agencies in the world for basic and clinical research. Most of the top 10 institutions and funding agencies are based in the USA. In addition to government fundings, pharmaceutical companies such as Bristol Myers Squibb and Merck Company also play a significant role in drug development and research. Regarding the top 10 co-cited references, six of them are from the USA, and one is a collaborative effort between the USA and the UK. USA scholars primarily publish clinical research and clinical guidelines, which enables their work to be widely disseminated and cited. China produces a considerable amount of research papers, but the citation rate is relatively low. The reasons behind this include language barriers, inadequate funding and support from the government or pharmaceutical industries, limiting large-scale clinical trials in some areas. As a result, researchers may prioritize conducting meta-analyses and case reports that have less impact on the scientific community compared to original research articles and clinical trials, thereby leading to a decreased citation rate. To address this, Chinese authorities should allocate more resources towards conducting original research articles and large-scale clinical trials to potentially increase the impact of research findings and contribute to the advancement of this research areas.

We also found that international collaboration has been increasing over time, with collaboration between the USA and France being particularly strong. This is an encouraging trend as international collaboration can lead to the sharing of knowledge and expertise, resulting in a better understanding of scientific research. China’s high publication output in the field of immunotherapy and irAEs indicates a growing interest and investment in research, which presents valuable opportunities for collaboration with researchers around the world. However, there may be limitations to China’s collaboration with other countries due to language barriers and a lack of established relationships with researchers in other countries. Additionally, China faces funding and pharmaceutical development challenges, with many local companies focusing on generic drugs rather than original products. Nonetheless, China has a large population of cancer patients, making it an attractive market for the development of new therapies. By collaborating more closely with other countries, we can effectively facilitate knowledge exchange and sharing, while simultaneously driving innovation in the healthcare industry. This can lead to a better understanding of diseases, drugs, and treatment options, ultimately resulting in improved patient care.

Our analysis further identified the top journals and institutions that have contributed to research on ICIs associated rheumatic irAEs. The top ten most central journals in this field were identified, and the institutions with the highest number of publications were also analyzed. Our findings can help researchers identify the most relevant journals and institutions to publish their research and collaborate with other researchers in this field. Additionally, researchers can subscribe to these journals to stay up-to-date with the latest developments in the field.

We also analyzed the keywords used in these publications. Analyzing the keywords used in publications can offer information about the research topics and methodologies of the publications, and analysis of keywords’ co-occurrence can often detect research hotspots and directions in the research field. By generating a network visualization map for keywords with the co-occurrence value greater than 20 times, we found that the most frequently used keywords can be broadly categorized into three sections: ICIs and their tumor indications, the mechanisms and efficacy of ICIs, and the adverse reactions caused by ICIs. As time progressed, researchers gained a deeper understanding of ICIs beyond their development as cancer treatments, with emphasis on their mechanisms, efficacy, and adverse reactions. The research hotspots included autoimmune hypophysitis, induced sarcoidosis, safety, inflammatory arthritis, and preexisting autoimmune conditions. These hotspots provide insights into the current research focus and highlight areas that require further investigation.

However, it is also important to acknowledge potential limitations and biases of our study. Firstly, this study solely relied on literature from the WoSCC database, which may have resulted in the exclusion of publications from other sources. Secondly, the study overlooked non-English articles, as it is focused only on English-language publications. These factors should be considered while interpreting the results. Another limitation of this study could be the lack of attention paid to the quality of the publications. While bibliometric methods can provide a comprehensive analysis of the literature, they do not take into account the quality of the research. Future studies could consider incorporating quality assessment tools to better understand the impact and significance of the research conducted on ICIs-associated rheumatic irAEs. Upon reviewing the literatures, we found that majority of them focused solely on describing the phenomenon of ICIs associated rheumatic irAEs, with a lack of comprehensive research on the underlying mechanisms, risk factors, and prevention strategies. We believe that delving deeper into these areas will significantly contribute to enhancing the prognosis of cancer patients. Despite these limitations, our study provides valuable insights that can significantly support researchers in their decision-making process when it comes to selecting suitable journals and institutions to publish their research or collaborate with. Additionally, this study sheds light on the current research hotspots and directions within the specific field of research being examined. These findings can contribute to enhancing the quality and impact of researchers’ work, as well as facilitating fruitful collaborations and identifying potential areas for future exploration.

Overall, our study is the first to use bibliometric methods such as CiteSpace and VOSviewer to comprehensively evaluate the current state and progress, as well as research trends and contributions in the field of ICI-induced rheumatic irAEs, which represents a new area of rheumatology that calls for urgent attention, research, and collaboration. Given the rapidly increasing use of ICIs in cancer treatment, rheumatologists can play a central role in the management of irAEs in the future, and multidisciplinary collaboration is essential to improving their recognition and outcomes. Future efforts should focus on developing classification criteria and guidelines for the diagnosis and management of rheumatic irAEs; conducting prospective studies to better estimate the incidence and prevalence of these events and understand their risk factors and patterns of onset, resolution, and persistence; investigating the immunopathogenic mechanisms involved, including the roles of different immune checkpoints, autoantibodies, and cytokines in their development and persistence; and identifying biomarkers for prediction and monitoring of these events based on their immunological and clinical features. These future directions are crucial to promoting better care for cancer patients treated with ICIs and advancing the field of rheumatology.

## 5 Knowledge base

ICIs have emerged as a promising treatment option for various types of cancers. However, their use has been associated with various irAEs, including rheumatic irAEs, which is a growing concern now. Compared to other immune AEs, rheumatic irAEs typically involve the joints, muscles, and other connective tissues. While arthralgia and myalgia are common rheumatic irAEs reported in clinical trials, other case series and reports have described a wider spectrum of *de novo* rheumatic and systemic manifestations that mimic classic rheumatic diseases and can occur with ICIs (25, 26). These include polymyalgia rheumatica (PMR)-like syndromes and inflammatory arthritis syndromes, which are often encountered clinical presentations (13, 27, 28). Systemic manifestations have also been reported, including sicca syndrome, sarcoidosis, and all vessel-sized vasculitis (29–35). Interestingly, autoantibodies are often absent or at low titre in these patients (11). While these rheumatic irAEs can occur across all classes of ICI, they are most frequently and severely associated with combination treatments and may be linked with other organ-specific irAEs. Treatment of rheumatic irAEs generally depends on the CTCAE grade of the events, and may involve discontinuing ICI therapy, administering steroids or immunosuppressive agents, including biologics, and referral to a rheumatologist for specialized care (11). Careful evaluation and monitoring of treatment response and potential side effects are critical as these patients may be at higher risk for infections and other complications.

Currently, the underlying immunopathogenic mechanisms of rheumatic irAEs are not clear and several possible mechanisms have been proposed. One might involve generalized immune activation, due to checkpoint neutralization, which might result in inflammatory reactions in multiple organ systems, including the musculoskeletal system (9). It is reported that several primary immunodeficiencies associated with prominent autoimmune manifestations are linked to dysfunction or underexpression of CTLA4 (36). T cell exhaustion, which correlates with a state of low disease activity in some autoimmune diseases, such as anti-nuclear cytoplasmic antibody (ANCA)-associated vasculitis, is also a possible explanation for this mechanism (37). A study found increased production of inflammatory cytokines such as IL-17 following treatment with ICIs, as well as the effectiveness of cytokine targeting (especially by TNF inhibitors) at treating immune-related side effects further supports it (38–40). Another proposed mechanism of rheumatic irAEs is direct off-target effects of checkpoint inhibitors (9). ICIs may have off-target effects on cells other than T cells, including natural killer cells, dendritic cells, and macrophages, leading to autoimmune inflammation and rheumatic irAEs. However, the precise mechanisms by which these effects occur are not yet clear. The third proposed mechanism of rheumatic irAEs is epitope spreading (41). The diversity of T-cell receptor repertoires and the heterogeneity of tumor-associated antigens make it challenging for the immune system to recognize and eliminate tumors. However, ICIs can induce epitope spreading, which is the diversification of the epitope-specificity of T-cells. The new targets acquired by the T cells can include new antigens on self-proteins, leading to autoimmune inflammation and rheumatic irAEs (41). In addition, molecular mimicry is also suggested as a possible mechanism of ICIs-associated rheumatic irAEs. Some viral and bacterial antigens share sequence or conformational similarity with host antigens. The immune system, in response to infections, may produce antibodies and T cells that cross-react with host antigens, leading to autoimmune inflammation and rheumatic irAEs. Pre-existing asymptomatic autoimmunity is another possible mechanism involving ICIs that mediate immune activation in asymptomatic or pauci-symptomatic autoimmunity (9). However, against this argument is the fact that most patients with rheumatic irAEs test negative for serum autoantibodies. To date, rheumatic irAEs have not been thoroughly investigated for signature autoantibodies, HLA associations, or biomarkers of idiopathic disease. However, as investigations are ongoing, there is a desperate need to study immunopathogenic mechanisms for most irAEs as such investigations are far from complete.

## Data availability statement

The raw data supporting the conclusions of this article will be made available by the authors, without undue reservation.

## Author contributions

LZ and QZ participated in the research design. LZ and KC participated in the bibliometric analysis. GM, LZ and QZ drafted the manuscript, QZ revised and polished the manuscript. All authors contributed to the article and approved the submitted version.
