# Supplementary material for: Bibliometric analysis of rheumatic immune related adverse events associated with immune checkpoint inhibitors
Source: Front Immunol. 2023 Oct 6;14:1242336. doi: 10.3389/fimmu.2023.1242336 (PMC10587544; doi:10.3389/fimmu.2023.1242336)
Supplement: Supplementary file 1 [file Image_1.pdf]

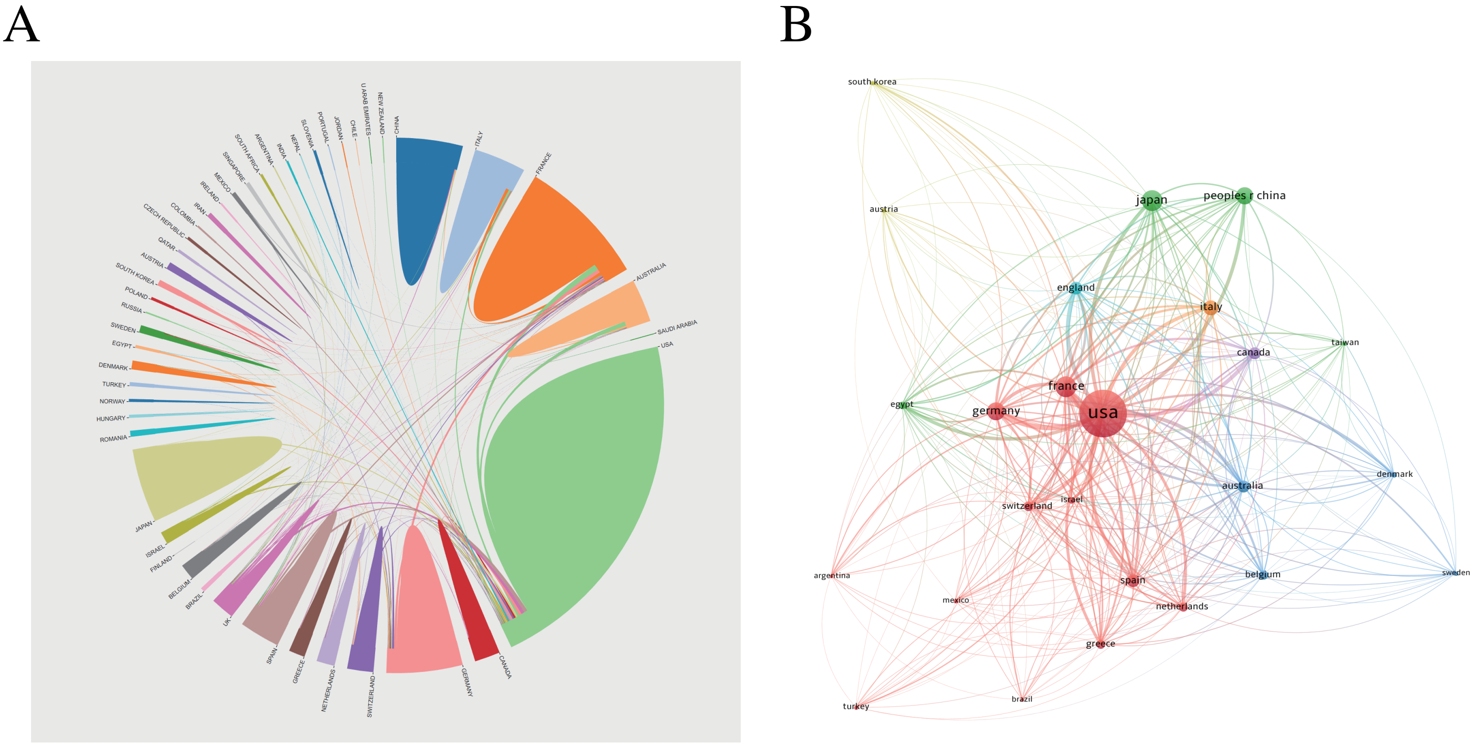

Supplementary Figure 1. (A) International cooperation among countries/regions in the field. In the network visualization, colors are used to represent the countries, and lines indicate international cooperation. The thickness of the line reflects the level of cooperation, with thicker lines indicating closer cooperation. (B) Co-citation analysis of countries/regions using VOSviewer. In the network visualization, nodes represent countries/regions, and their size reflects the number of publications. Lines between nodes indicate the citation relationship between countries/regions.
