# Supplementary material for: Bibliometric analysis of rheumatic immune related adverse events associated with immune checkpoint inhibitors
Source: Front Immunol. 2023 Oct 6;14:1242336. doi: 10.3389/fimmu.2023.1242336 (PMC10587544; doi:10.3389/fimmu.2023.1242336)
Supplement: Supplementary file 2 [file Image_2.pdf]

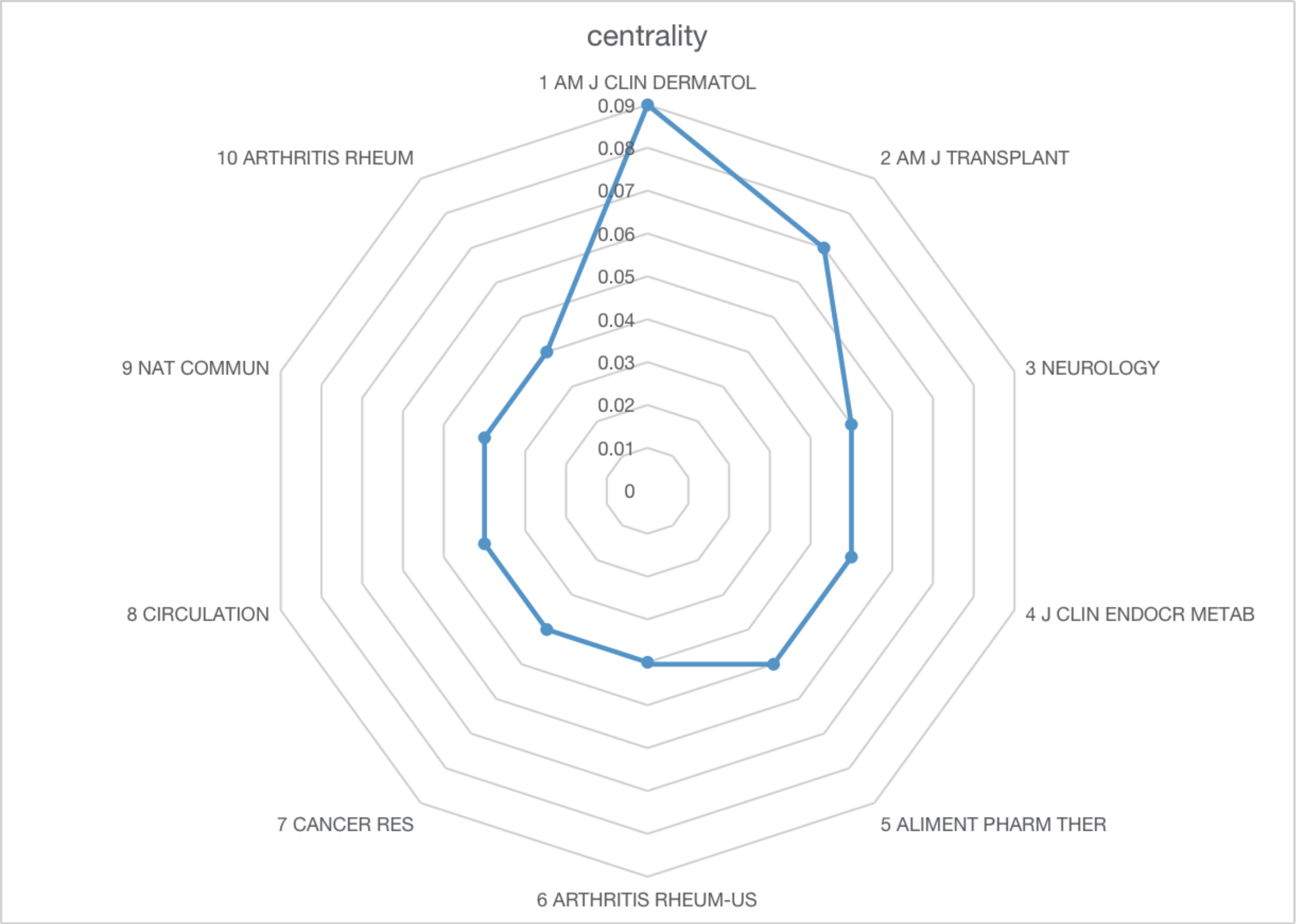

Supplementary Figure 2. The 10 most central journals ranked by centrality in the field of ICIs associated rheumatic irAEs using CiteSpace.
