# Supplementary material for: Bibliometric analysis of rheumatic immune related adverse events associated with immune checkpoint inhibitors
Source: Front Immunol. 2023 Oct 6;14:1242336. doi: 10.3389/fimmu.2023.1242336 (PMC10587544; doi:10.3389/fimmu.2023.1242336)
Supplement: Supplementary file 3 [file Image_3.pdf]

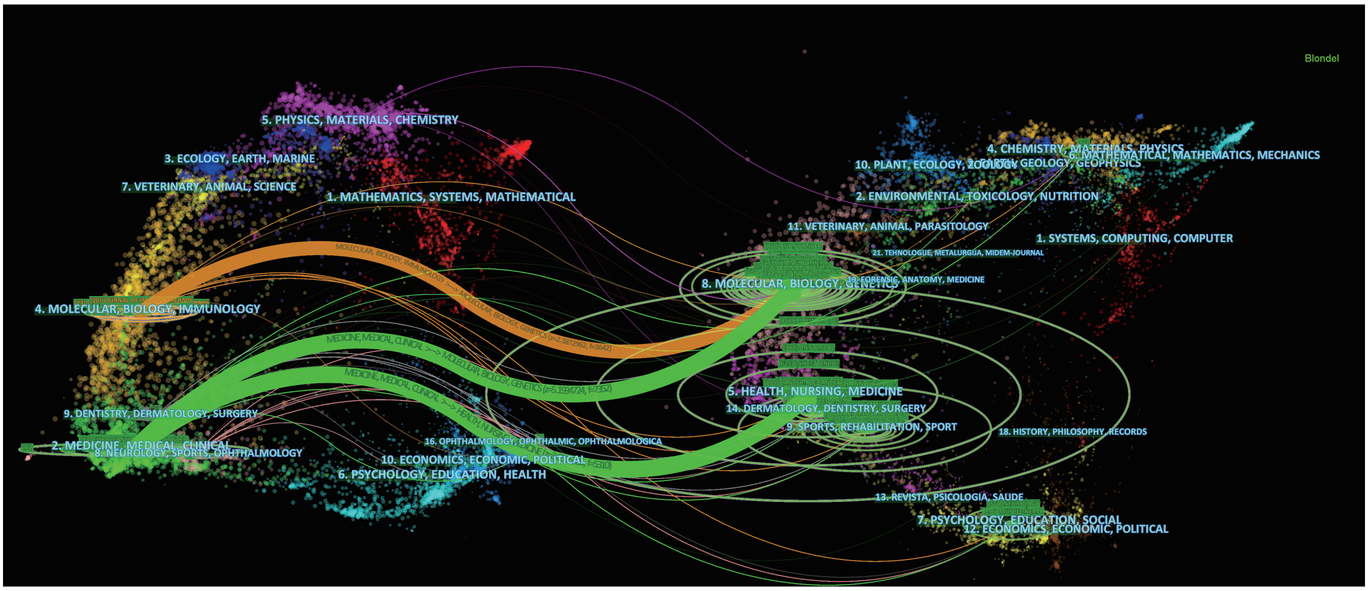

Supplementary Figure 3. Dual-map overlay of citing and cited journals in the field of ICIs associated rheumatic irAEs using CiteSpace. The left side of the visualization shows citing journals, while the right side shows cited journals. Reference paths are indicated by different colored lines.
