# Supplementary material for: Bibliometric analysis of rheumatic immune related adverse events associated with immune checkpoint inhibitors
Source: Front Immunol. 2023 Oct 6;14:1242336. doi: 10.3389/fimmu.2023.1242336 (PMC10587544; doi:10.3389/fimmu.2023.1242336)
Supplement: Supplementary file 4 [file Image_4.pdf]

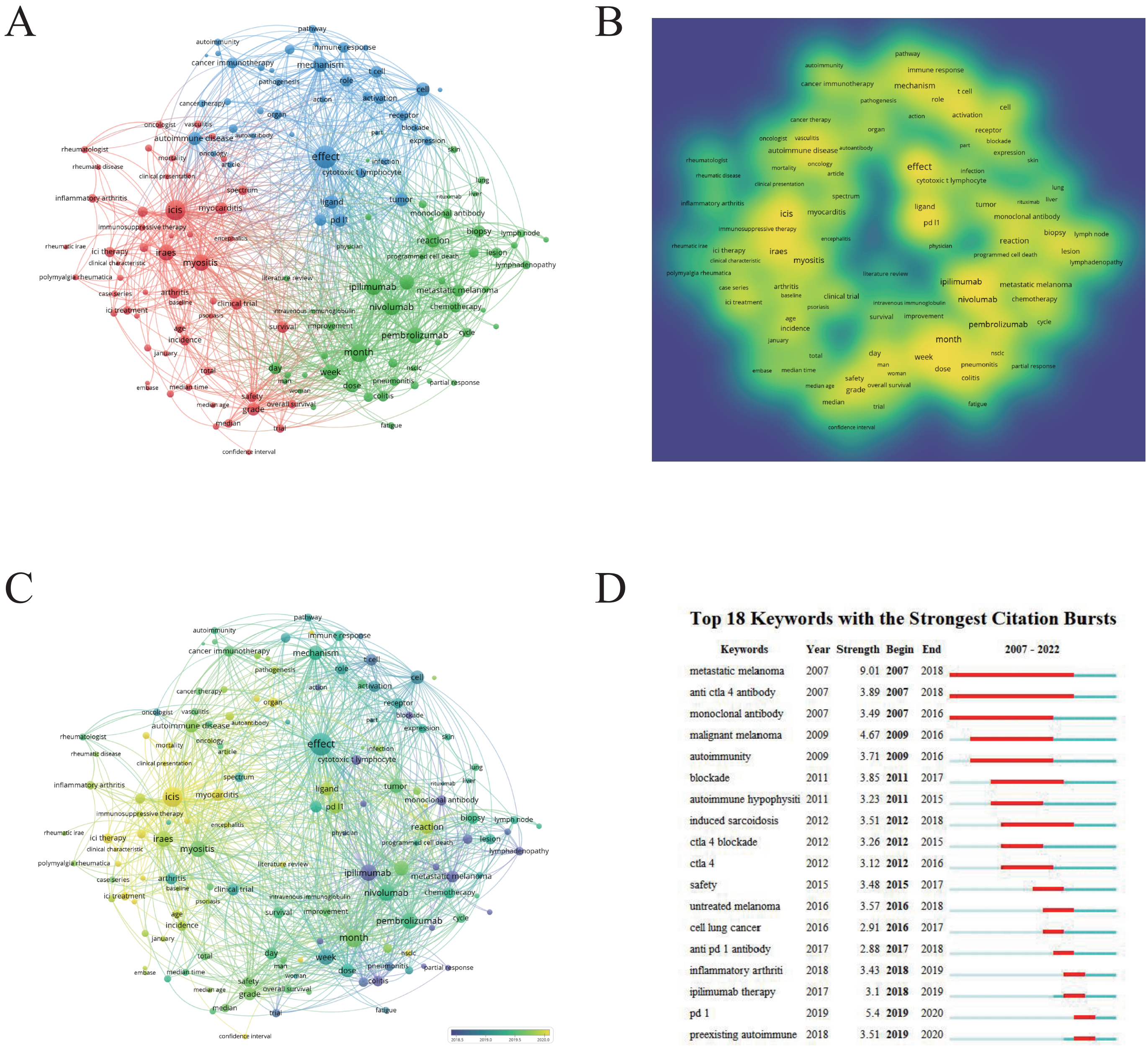

Top 18 Keywords with the Strongest Citation Bursts

Keywords	Year	Strength	Begin	End	2007 - 2022
metastatic melanoma	2007	9.01	**2007**	2018	
anti ctla 4 antibody	2007	3.89	**2007**	2018	
monoclonal antibody	2007	3.49	**2007**	2016	
malignant melanoma	2009	4.67	**2009**	2016	
autoimmunity	2009	3.71	**2009**	2016	
blockade	2011	3.85	**2011**	2017	
autoimmune hypophysiti	2011	3.23	**2011**	2015	
induced sarcoidosis	2012	3.51	**2012**	2018	
ctla 4 blockade	2012	3.26	**2012**	2015	
ctla 4	2012	3.12	**2012**	2016	
safety	2015	3.48	**2015**	2017	
untreated melanoma	2016	3.57	**2016**	2018	
cell lung cancer	2016	2.91	**2016**	2017	
anti pd 1 antibody	2017	2.88	**2017**	2018	
inflammatory arthriti	2018	3.43	**2018**	2019	
ipilimumab therapy	2017	3.1	**2018**	2019	
pd 1	2019	5.4	**2019**	2020	
preexisting autoimmune	2018	3.51	**2019**	2020	

Supplementary Figure 4. Visualization analysis of keywords in the field of ICIs associated rheumatic irAEs. (A) In this network map, closely related keywords are grouped and labeled with the same color, and three clusters are displayed. (B) VOSviewer is used to visualize keyword density. (C) A time-lapse visualization of keyword popularity over time is shown, with purple representing earlier dates and yellow representing more recent dates. (D) List of the top 18 keywords with the strongest citation bursts.
